# Brine Pretreatment Improves Ion-Exchange Resin Regeneration with Associated Physicochemical Changes

**DOI:** 10.1021/acsomega.6c07602

**Published:** 2026-09-30

**Authors:** Shin-Yong Yeoh, Kher-Wei Lai, Swee-Yong Pung, Kun-Yi Andrew Lin, Muhammad Qasim Ali, Shi Yu Khor, Fei-Yee Yeoh

**Affiliations:** † Food Technology Division, School of Industrial Technology, 26689 Universiti Sains Malaysia, Building G07, Persiaran Sains, Pulau Pinang 11800, Malaysia; ‡ School of Materials & Mineral Resources Engineering, Universiti Sains Malaysia, Nibong Tebal, Penang 14300, Malaysia; § Department of Environmental Engineering, 34916 National Chung Hsing University, 250 Kuo-Kuang Road, Taichung 402, Taiwan; ∥ Faculty of Industrial Sciences and Technology, 65240 Universiti Malaysia Pahang Al-Sultan Abdullah, Gambang, Pahang 26300, Malaysia; ⊥ Pusat Perniagaan Setia Sentral, Tekno Impact PLT, 1, First Floor, Jalan Setia Sentral, Bukit Mertajam, Penang 14000, Malaysia

## Abstract

Regeneration efficiency governs the long-term performance and sustainability of ion-exchange processes, yet resin fouling and limited accessibility of exchange sites can restrict regeneration effectiveness and ion transport. This study investigates controlled brine pretreatment as a regeneration-enhancement strategy before conventional acid–base regeneration. Under optimized conditions (5% NaCl, 1:1 resin-to-brine ratio, and 55 °C), pretreated resins achieved approximately 306% higher Ca^2+^ removal and 222% higher Mg^2+^ removal than untreated regenerated resins. In individual column runs, breakthrough analysis showed a longer resin utilization period for the pretreated resin, with the exhaustion time increasing from 49 to 69 min. The times to reach C/C_0_ values of 0.90 and 0.95 also increased from 43 to 62.8 min and from 45.1 to 65.0 min, respectively. SEM showed no obvious surface-associated deposits on the pretreated resin, while FTIR analysis indicated minor spectral changes with characteristic resin functional groups largely retained. Particle-size analysis showed a shift toward smaller particle sizes after brine pretreatment. These physicochemical changes may influence exchange-site accessibility and ion transport. ICP–MS analysis further showed that approximately 65.6% of Ca^2+^ and 66.4% of Mg^2+^ were displaced during the brine pretreatment stage before subsequent acid–base regeneration. The findings provide indirect physicochemical evidence linking controlled brine pretreatment to observed changes in resin characteristics and regeneration behavior.

## Graphical abstract

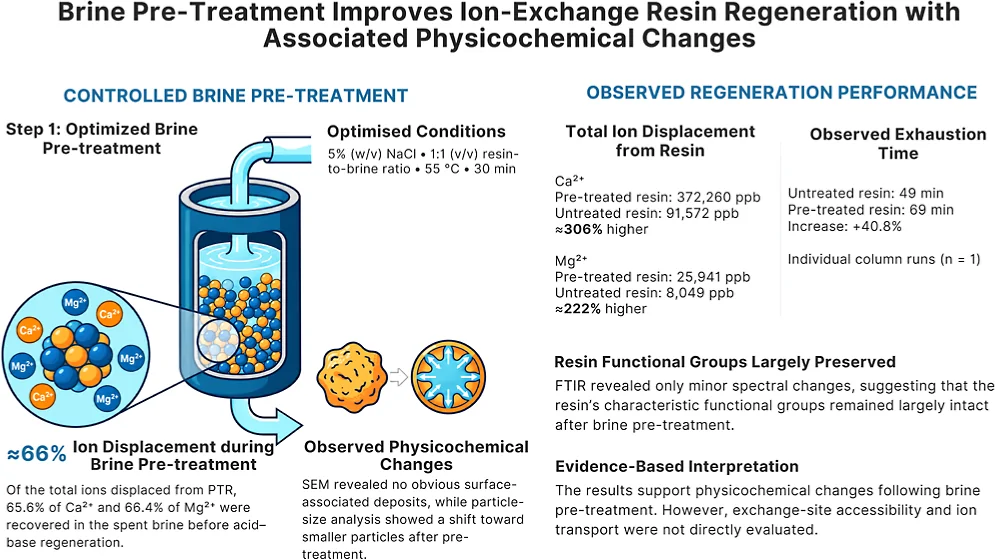

## 1 Introduction

Freshwater availability is limited, and less than 1% of the global freshwater is readily accessible for human use. Groundwater, a major source, often contains elevated levels of hardness, primarily because of calcium (Ca^2+^) and magnesium (Mg^2+^) ions. Hardness above 200 mg/L is generally undesirable, and levels above 300 mg/L can lead to serious scaling, reduced heat transfer efficiency, and pipeline blockages.^1^ Temporary hardness (from bicarbonates) can be removed by boiling, whereas permanent hardness (from sulfates and chlorides) requires more advanced technologies such as ion exchange or chemical softening.^2^ If these ions are not removed, they can precipitate as mineral scales on pipes and boilers, impairing thermal efficiency and increasing maintenance demands.^3^ Therefore, the development of efficient and sustainable water softening technologies remains important for both domestic and industrial applications.

Several methods have been used for hardness removal, including nanofiltration, electro-deionization, adsorption, microbial treatment, and lime–soda softening.^1^ Among these, ion exchange is widely recognized as a practical and cost-effective approach due to its high efficiency, regenerability, simple operation, and low sludge production.^4^ Ion-exchange resins (IERs) are categorized into cation and anion types. Cation resins are either strongly or weakly acidic, while anion resins are strongly or weakly basic, depending on their functional groups. Mixed-bed resins combine both types to remove cations and anions simultaneously.^5^ In practice, systems using strong-acid cation (SAC) and strong-base anion (SBA) resins are commonly applied to remove Ca^2+^ and Mg^2+^ and produce high-purity water.^4^ The ion-exchange process is governed by electrostatic interactions and ion-exchange equilibrium and is influenced by resin structure, cross-link density, and hydration behavior.^6,7^ Recent developments, such as shallow-shell technology (SST), have improved ion-exchange efficiency by shortening diffusion pathways and reducing regenerant demand while maintaining hardness removal. SST anion resins also demonstrate rapid nitrate uptake and efficient regeneration using low-acid solutions, even in the presence of competing ions.^8,9^

Beyond conventional IERs, ion-exchange membranes (IEMs) are increasingly applied in water purification, wastewater treatment, desalination, and resource recovery. IEMs contain fixed charged functional groups that enable selective ion transport, with performance governed by the membrane structure, ion-exchange capacity, swelling, and ion selectivity. Recent developments in cation- and anion-exchange membranes have focused on improving these properties for applications such as electrodialysis, diffusion dialysis, and selective ion recovery.^10,11^ These developments highlight the broader importance of the material structure and ion-transport characteristics in determining the performance of ion-exchange systems.

A resin is considered depleted once its exchange capacity is exceeded, as indicated by rising effluent conductivity, and is regenerated using a high-concentration regenerant, typically a mineral acid or concentrated salt solution, to displace retained ions.^5^ However, the sustainability of ion-exchange softening is increasingly limited by the environmental impact of regeneration. Conventional use of sodium chloride (NaCl), hydrochloric acid (HCl), and sodium hydroxide (NaOH) produces highly saline waste brine, increases chemical consumption, and accelerates resin fouling.^12–14^ Improving regeneration efficiency is essential for enhancing exchange-site utilization and process performance while simultaneously reducing regenerant consumption, operational costs, and associated environmental impacts.^5^ Frequent regeneration increases chemical consumption, waste production, and resin fouling, reducing sustainability and increasing operational costs.^15^ Although alternative regeneration aids such as alkalis, surfactants, or oxidants have been explored, these methods are expensive and are not always effective against irreversible fouling.^16^ Reducing regeneration frequency has been proposed to alleviate these issues.^7^ Consequently, there remains a need for regeneration strategies that improve ion accessibility, regeneration efficiency, and operational performance while reducing chemical consumption. Despite advances in resin design and process optimization, regeneration remains limited by high regenerant consumption, fouling, and reduced accessibility of exchange sites.

Our previous study optimized the brine pretreatment conditions for ion-exchange resin (IER) regeneration by evaluating the effects of treatment temperature, NaCl concentration, and resin-to-brine ratio.^17^ However, process optimization alone does not explain how brine pretreatment influences resin properties and subsequent regeneration performance. Although several studies have focused on optimizing regenerant concentration, contact time, and operating conditions, the physicochemical changes induced by brine pretreatment and their implications for regeneration efficiency remain insufficiently understood. Therefore, this study investigates the effects of controlled brine pretreatment on the physicochemical characteristics and demineralization performance of spent ion-exchange resins. By integrating SEM, FTIR, particle size distribution, conductivity breakthrough analysis, and ICP–MS measurements, the relationship between resin physicochemical changes and regeneration performance was evaluated. The study further examined the potential of low-concentration NaCl pretreatment to facilitate ion displacement before conventional acid–base regeneration and assessed the extent of this displacement and its potential implications for regenerant demand following further process optimization. It is hypothesized that brine pretreatment modifies resin physicochemical properties in ways that may influence exchange-site accessibility and subsequent regeneration performance. The findings contribute to a better understanding of regeneration enhancement and support the development of more efficient ion-exchange water treatment processes.

## 2 Materials and Methods

### 2.1 Materials

Spent mixed-bed IERs were obtained from a local industrial facility in Penang, Malaysia, consisting of an approximate 2:1 ratio of cation to anion beads, with a uniformity coefficient of 1.697, a dominant particle size of 0.850 mm (sieve retention), and a moisture content of 68–71%, as determined by oven-drying at 120 °C. Sodium chloride (NaCl, ≥99.5% purity; insoluble matter ≤0.005%) and sodium hydroxide (NaOH, ≥98%) were purchased from Merck (Darmstadt, Germany), while hydrochloric acid (HCl, ∼37% w/w, analytical grade) was used to prepare regenerant solutions. All other reagents were of analytical grade (Sigma-Aldrich, Selangor, Malaysia).

### 2.2 Preparation of Resins for Analysis

Spent mixed-bed IERs were obtained from an industrial source and classified into four groups: untreated resin (UT), resin with optimized brine pretreatment parameters (PT), and their corresponding regenerated forms (UTR and PTR). UT and PT resins were characterized for physicochemical properties, including moisture content, particle size distribution, and Fourier transform infrared (FTIR) analysis, while PT resin was additionally examined using scanning electron microscopy (SEM). The regenerated resins (UTR and PTR) were evaluated for demineralization performance and ion removal efficiency.

### 2.3 Moisture Content Analysis

Moisture content was determined using a modified ASTM D 2187-94 (Water Retention Capacity Test).^18^ UT resin (60 mL) was soaked in 600 mL of DI water for 8 h to achieve full swelling. A 10 mL portion was mixed with 90 mL of DI water, and the resin weight was calculated as

weightof10mLofresin(g)=weightof90mLofDIwaterwithresin(g)−weightof90mLofDIwater(g)

The remaining 60 mL of swollen resin was filtered, placed in a Petri dish, and dried in a Memmert oven at 120 °C. Every hour for 6 h, 10 mL of resin was removed, cooled for 10 min, and weighed. Moisture content was calculated as

$$moisture\;content\;\left(\%\right)=\frac{swollen\;resin\;weight\;\left(g\right)-dried\;resin\;weight\;\left(g\right)}{swollen\;resin\;weight\;\left(g\right)}\times 100\%$$

For volumetric analysis, 10 mL of dried resin was soaked in 90 mL of DI water, and the total volume was recorded after 1 h. Volumetric swelling was calculated as

$$volumetric\;swelling\;\left(\%\right)=\frac{resin\;volume\;after\;soaking\;\left(mL\right)-initial\;volume\;of\;dried\;resin\;\left(mL\right)}{initial\;volume\;of\;dried\;resin\;\left(mL\right)}\times 100\%$$

### 2.4 FTIR Spectroscopy

FTIR spectra were recorded using a PerkinElmer 1750 spectrometer (Waltham, USA) at room temperature, scanning from 500–4000 cm^–1^ with 4 scans.^19^ Resin samples (2 mg) were mixed with KBr (200 mg for single resin; 20 mg for mixed resin), ground, and pressed into 13 mm pellets at 400 psi for 2 min using a hydraulic press.

### 2.5 Preparation of PT Resin with Optimized Parameters

The resin prepared using all optimized brine pretreatment was coded as PT. UT resin was soaked in DI water overnight and subsequently oven-dried at 120 °C for 6 h using a Memmert oven. The resin was subjected to batch brine pretreatment using 30 mL of 5% (w/v) NaCl solution per 30 mL of resin (1:1, v/v) at 55 °C. Brining was performed for 30 min on a platform shaker at 400 rpm, followed by 10 min of settling. The spent brine was collected, and the resin was rinsed six times with 500 mL of DI water for 10 min per cycle, followed by 3 min of settling and decantation after each rinse. The resin was subsequently soaked overnight in 500 mL of DI water and subjected to four additional rinses, each using 500 mL of DI water. For final conditioning, the resin was immersed in 500 mL of tap water overnight.^17^

### 2.6 SEM

The surface morphology of dried and swollen PT resins was examined using a Hitachi TM3000 Tabletop SEM (Tokyo, Japan).^17^ Swollen PT resins were prepared by soaking the dried PT resin in DI water overnight. Before SEM analysis, samples were sputter-coated with Au/Pd at 20 mA. Imaging was conducted in analytical mode at 20 kV with magnifications ranging from 40× to 2000×.

### 2.7 Particle Size Analysis

Particle size distributions of UT and PT resins were determined according to ASTM D 2187-94 using U.S. Standard Sieves (No. 8–100, excluding No. 16 and 20).^18^ The cumulative percentage retained was plotted against sieve opening diameter (mm) to generate Gaussian distribution curves. The uniformity coefficient (UC) was calculated as

$$uniformity\;coefficient\;\left(UC\right)\;after\;brine\;pre‐treatment=\frac{sieve\;size\;\left(mm\right)\;retained\;40\%\;of\;the\;sample}{sieve\;size\;\left(mm\right)\;retained\;90\%\;of\;the\;sample}$$

### 2.8 Preparation of UTR and PTR Resins

The regeneration of UTR and PTR resins was carried out by backwashing, separation, and chemical treatment to obtain UTR and PTR samples, respectively.^20^ The anion resin was regenerated using a 1% (w/v) NaOH solution, prepared by dissolving 1 g of NaOH in a 100 mL volumetric flask and topping up with DI water. The cation resin was regenerated by using a 4% (w/v) HCl solution prepared by diluting 10.81 mL of 37% HCl to 100 mL with DI water. For backwashing, 700 mL of DI water was introduced through the base of a chromatography column (25 mm inner diameter, 300 mm length), allowing the resin to fluidize and separate into three distinct layers: anion, mixed, and cation. The anion and cation layers were collected using suction pipettes (30 and 60 mL, respectively), while the mixed layer was discarded. The separated anion and cation resins were then treated with 16 mL of 1% NaOH and 36 mL of 4% HCl, respectively, and agitated on a shaker at 400 rpm for 50 min, followed by 5 min of settling. After regeneration, the resins were blow-dried for 40 min and manually stirred in a beaker for 3 min to ensure homogeneity. The spent regenerants (4% HCl and 1% NaOH) and spent NaCl brine were collected and analyzed for Ca, Mg, and iron (Fe) concentrations using a PerkinElmer NexION 300 Inductively Coupled Plasma Mass Spectrometer (ICP–MS) (Waltham, USA). The detection limits for Ca, Mg, and Fe were 1.101, 9.924, and 0.619 ppb, respectively. A total of 90 mL of the UTR and PTR resins was used for this analysis.

### 2.9 Analysis of Brine Pretreatment Efficiency

To evaluate brine pretreatment efficiency and demineralization performance, tap water was first filtered through a sand filter, followed by activated carbon, resulting in influent water with a conductivity of 74.80 μS cm^–1^. The filtered water was then passed through an ion-exchange resin column (length 25 mm, inner diameter 39 mm) packed with a 2:1 volumetric mixture of cation and anion resins. During the demineralization process, effluent conductivity was continuously measured using a Hanna HI2003 electrical conductivity meter (Providence, USA) until it reached the influent value, indicating resin exhaustion. The influent water contained Ca^2+^ at 6.12 ± 0.08 ppm and Mg^2+^ at 0.63 ± 0.02 ppm, which were used as baseline concentrations for calculating removal efficiencies. Identical batches of influent water were used for paired UTR and PTR experiments to ensure comparability. Conductivity breakthrough experiments were conducted under identical operating conditions, while ICP–MS analyses were performed in triplicate and reported as mean ± standard deviation. Conductivity breakthrough experiments for UTR and PTR were each conducted as individual column runs (*n* = 1). Therefore, the breakthrough profiles represent experimentally observed individual runs rather than mean values from replicate experiments.

## 3 Results and Discussion

### 3.1 Moisture Content Analysis

The moisture content of UT resin was 71% after 2 h of drying at 120 °C and decreased slightly to 68.01% after 5 h (data not shown), indicating that the drying conditions were sufficient to achieve a stable dry mass. This observation is consistent with a previous study reporting that mass loss below 120 °C in a strongly basic anion-exchange resin was associated with the evaporation of adsorbed water.^22^

The moisture content observed in this study is higher than typical values reported for SAC (53–58%) and SBA (59–60%) resins, which may be consistent with a relatively low cross-linking density. Resin water uptake is influenced by cross-linking density, functional groups, and ionic form, where lower divinylbenzene (DVB) content promotes greater swelling due to a more open polymer structure. Additionally, resins in the H^+^ form tend to retain more water than those in the Na^+^ form due to stronger hydration interactions.^5^ Resins with lower cross-linking density are advantageous for enhancing ion diffusion and exchange kinetics. However, they may also be more susceptible to swelling and mechanical instability.^3^ Previous studies have shown that high-moisture resins (∼75%) exhibit improved removal efficiency for organic contaminants and small charged species due to increased accessibility of active sites.^6,23^ The elevated moisture content observed in this study may be consistent with a more hydrated polymer network. However, its effects on ion transport and exchange-site accessibility were not directly measured.

### 3.2 Mixing Ratio of Resins

FTIR spectroscopy is a rapid and economical technique for detecting foulants, evaluating structural degradation or deformation of ion exchangers, and identifying the functional groups responsible for the adsorption of specific ions.^15^ In the present study, characteristic absorption bands were observed in the 1025–800 cm^–1^ region. The cation resin exhibited signature peaks at 1008 and 1037 cm^–1^ corresponding to sulfonic acid (−SO_3_H) groups, whereas the anion resin showed a distinctive peak at 889 cm^–1^ attributed to the −N^+^(CH_3_)_3_ functional group (Figure 1). Consistent with previous reports, the relative difference in peak heights between 1008 and 890 cm^–1^ provided an indicative measure of the mixing proportion.

**Figure 1:**
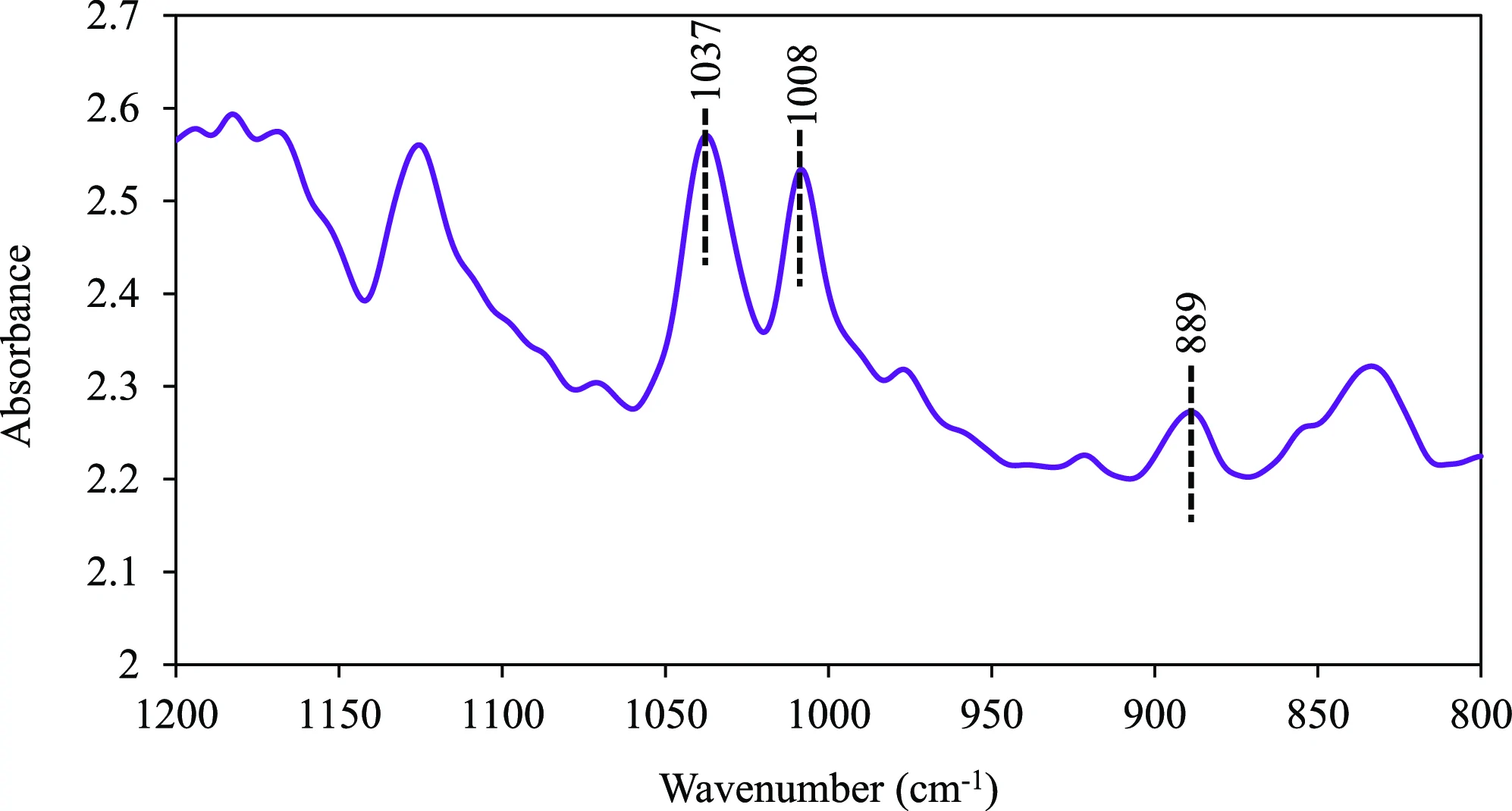
FTIR absorption spectra of a mixture of cation and anion IERs used in the present study.

For a known 1:1 (v/v) cation-to-anion mixture, Ghosh et al.^24^ reported an absorbance difference of 0.125% between the characteristic cation-resin (Indion-525) peaks at 1008 and 1037 cm^–1^ and the anion-resin (GS-300) peak at 890 cm^–1^. In contrast, the present study recorded an absorbance difference of 0.26% between the cation peaks (1008 and 1037 cm^–1^) and the anion peak (889 cm^–1^), which is approximately double that observed for the 1:1 mixture. This increase in peak-height difference reflects a higher proportion of cation resin relative to anion resin in the mixed-bed sample. Since the sulfonic acid (−SO_3_H) groups of the cation resin contribute stronger and more abundant vibrational signals in this region compared to the quaternary ammonium (−N^+^(CH_3_)_3_) groups of the anion resin, the relative absorbance difference is sensitive to compositional variation. The observed doubling, therefore, indicates an approximate 2:1 cation-to-anion mixing ratio in the batch sample, which aligns with commonly employed mixed-bed configurations reported in the literature.^25^

It should be noted, however, that this approach provides a semiquantitative estimate rather than an absolute quantification of the mixing ratio, as the small sample mass required for FTIR analysis may not fully reflect the bulk resin composition. Averaging multiple measurements improves reliability, as suggested in earlier studies. On the basis of these spectral observations and standardized calibration mixtures, a 2:1 cation-to-anion ratio was selected for subsequent experiments to ensure consistent ion-exchange performance during brine pretreatment and regeneration.

### 3.3 Morphology Changes

The mean diameter of hydrated UT resin reported previously was 576.67 μm, which decreased to 539.70 μm after drying.^17^ Surface examination revealed cracks, dents, fragments, and irregularities in both hydrated and dried states. These structural defects may be associated with operational and regeneration stresses and could affect long-term resin performance. For PT resin, the average particle size was 508.50 μm in the dried state (Figure 2b) and 588.40 μm in the swollen state (Figure 2a). There was a 2% increase in particle size for the swollen state and a 5.8% reduction for the dried state compared to UT resin, which may be associated with swelling in polar media and shrinkage upon drying.^26^ Notably, swollen and dried PT resin displayed cracking and surface fragmentation, which may be associated with osmotic effects during brine pretreatment. No obvious surface-associated deposits were observed on the PT resin after brine pretreatment. These findings highlight the importance of optimizing pretreatment and regeneration conditions to limit physical degradation while maintaining resin performance. The observed differences in surface deposits and morphology following pretreatment may be associated with differences in ion transport and exchange-site accessibility. However, these properties were not directly measured.

**Figure 2:**
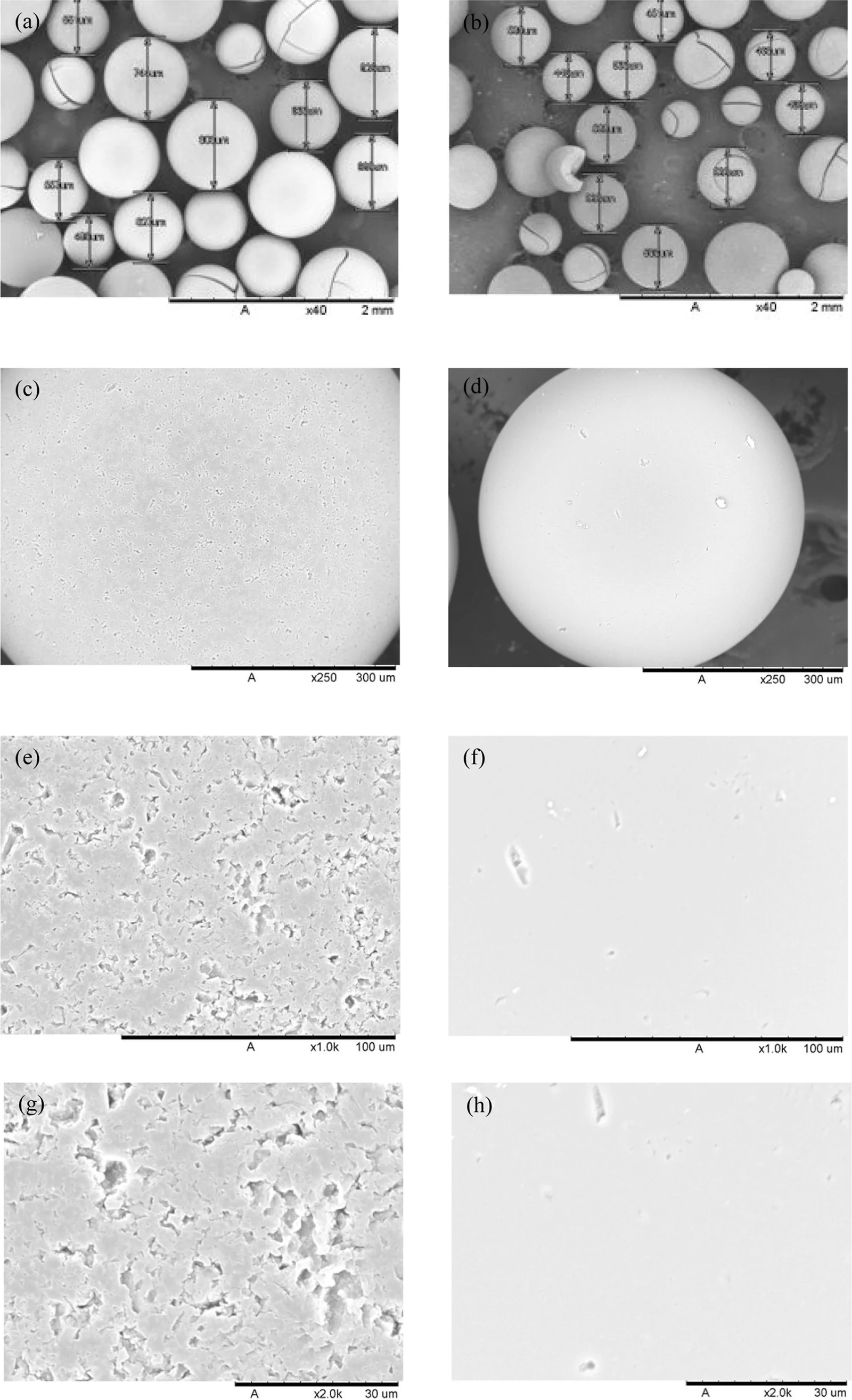
SEM micrographs of PT resin after brine pretreatment: (a,c,e,g) swollen state and (b,d,f,h) dried state at 40×, 250×, 1000×, and 2000× magnifications, respectively.

### 3.4 Particle Size Distribution

Figure 3a shows the particle size distribution of resin before and after brine pretreatment. The UT resin was primarily retained on the 0.850 mm sieve, whereas the PT resin shifted to the 0.727 mm sieve, reducing the average particle size after brine pretreatment. This leftward shift is consistent with a slight resin shrinkage resulting from the brine pretreatment. Previous studies have reported that smaller particles exhibit a higher surface area-to-volume ratio, which is advantageous for ion-exchange applications, as it increases the contact area and enhances ion diffusion and release rates.^27^ In addition, smaller resin particles have been reported to facilitate faster attainment of ion-exchange equilibrium due to shorter diffusion pathways, although they may reduce flow velocity through the column.^5^

**Figure 3:**
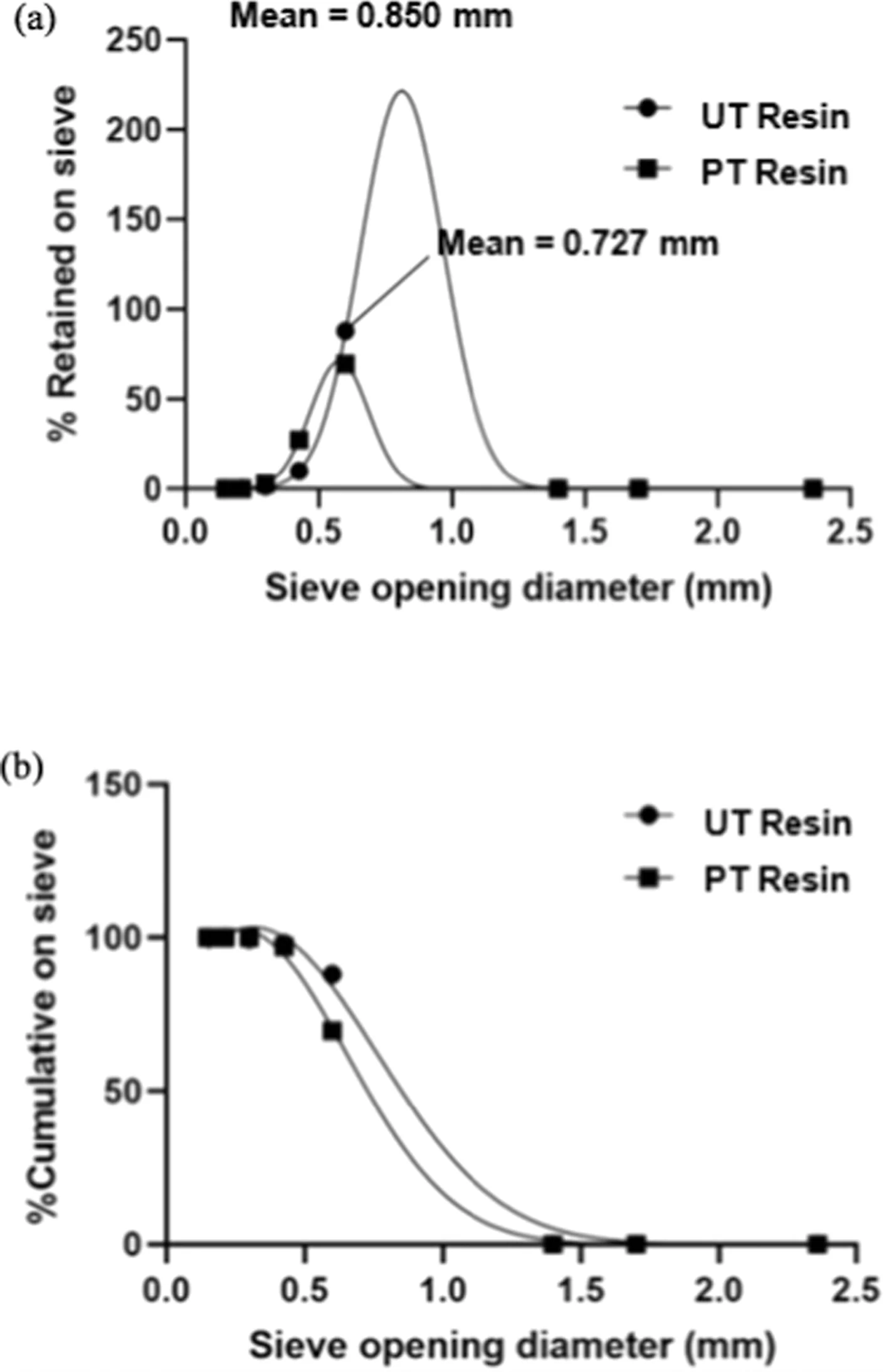
(a) Percentage of resin retained on each sieve plotted against sieve opening diameter (mm) for UT and PT resins; (b) cumulative percentage of resin retained versus sieve opening diameter (mm) for UT and PT resins.

Figure 3b presents the cumulative percentage retained and the calculated uniformity coefficient (UC), which increased slightly from 1.697 (UT resin) to 1.717 (PT resin), a change of only approximately 1%. This minimal shift suggests that brine pretreatment reduces average particle size but preserves the overall uniformity of the resin bead population, with both values falling within the optimal range for industrial ion exchange applications.^3^ Maintaining a consistent particle size distribution after treatment may contribute to more consistent hydraulic behavior during column operation.

### 3.5 FTIR Analysis

The FTIR spectra for UT and PT anion exchange resins are shown in Figure 4. The peaks at around 1640 cm^–1^ in both resins are attributed to O–H stretching and bending vibrations associated with water molecules.^28^ The peaks at approximately 1511 cm^–1^ in both resins are attributed to C=C stretching vibrations of benzene rings.^24^ New peaks were also observed in the PT anion resin, including bands at 1477 cm^–1^, attributed to C=C stretching and C–H asymmetric deformation, and at 1455 cm^–1^, corresponding to C–H deformation and CH/CH_2_ scissoring vibrations.^24^ The persistence of aromatic and quaternary ammonium bands after brine pretreatment indicates that the primary functional structure of the SBA was largely preserved after brine pretreatment, despite minor spectral changes associated with ion interactions and surface modifications. Similar preservation of characteristic functional groups following regeneration has been reported in FTIR analyses, indicating that observed spectral changes are primarily associated with ion binding and surface interactions, while the polymer backbone remains largely intact.^29^ The peaks at around 1384 cm^–1^ in both resins correspond to C–H bending and symmetric CH_3_ deformation vibrations, typically observed around 1380 cm^–1^.^15^ The strong peak around 889 cm^–1^ is attributed to single-bond N^+^(CH_3_)_3_ groups present in both UT and PT resins and is considered a characteristic signature of anion-exchange resins.^30^

**Figure 4:**
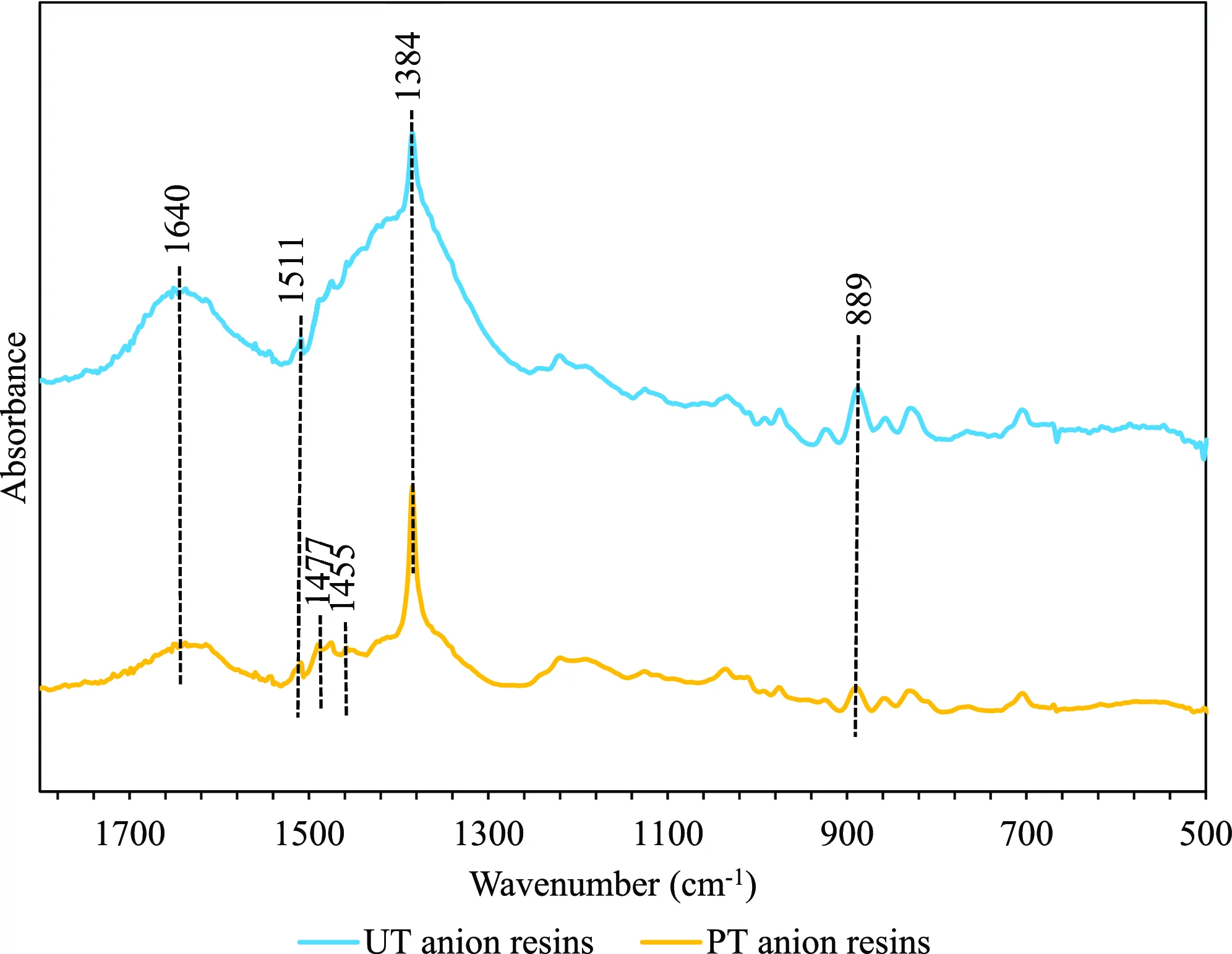
FTIR absorbance spectra of UT and PT anion resins.

Figure 5 presents the FTIR spectra and absorption peaks for UT and PT cation resins. The spectral region above 3000 cm^–1^ was omitted to eliminate the broad absorption band around 3400 cm^–1^ attributed to residual moisture present in both cation and anion resins, as this band does not correspond to intrinsic structural features of the resin matrix.^24^ Bands at around 1637 cm^–1^ are attributed to H_2_O bending vibrations.^31^ The peaks at around 1495 cm^–1^ correspond to aromatic C–C stretching, while the band at 1453 cm^–1^ is associated with CH_2_ deformation vibrations.^32^ The absorption band at 1412 cm^–1^ corresponds to the characteristic vibration of the methyl (−CH_3_) group.^32^ Characteristic peaks associated with sulfonic acid (−SO_3_H) groups were observed in both UT and PT resins, with minor shifts (e.g., 1172 to 1180 cm^–1^), suggesting preservation of the primary functional framework after brine pretreatment.^24,31^ The bands at 832 and 776 cm^–1^ (UT cation resin) and 833 and 776 cm^–1^ (PT cation resin) are associated with C–H out-of-plane deformation (benzene ring).^31^ After brine pretreatment, a slight peak shift from 1172 (UT cation resin) to 1180 cm^–1^ (PT cation resin) was detected, which may reflect changes in the local chemical environment of the resin functional groups. The disappearance of the 1212 cm^–1^ band in the PT resin may likewise reflect changes in surface-associated species accumulated during service. The FTIR results indicate that the characteristic functional groups of the resin were largely retained after brine pretreatment, while the observed spectral changes are consistent with changes in surface-associated species that may influence functional-group accessibility. These physicochemical observations are consistent with the differences in ion-exchange performance observed after pretreatment.

**Figure 5:**
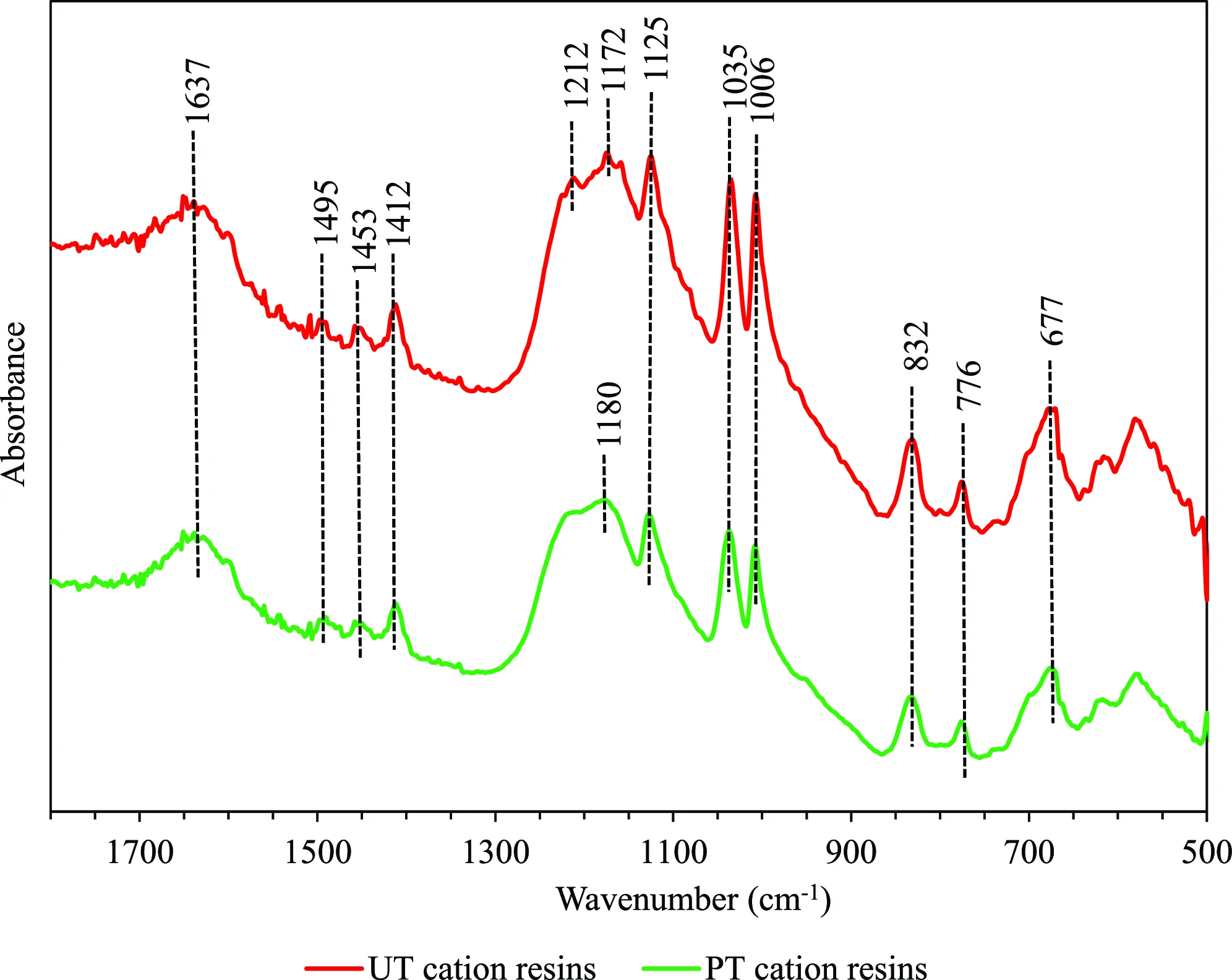
FTIR absorbance spectra of UT and PT cation resins.

### 3.6 Evaluation of Brine Pretreatment and Demineralization Performance

The observed demineralization behavior is consistent with the physicochemical changes following brine pretreatment, as evidenced by the SEM, FTIR, and particle-size analyses discussed earlier. These physicochemical changes, particularly the differences in surface-associated deposits and morphology observed by SEM, may influence ion transport and exchange-site accessibility. Regeneration performance was evaluated by monitoring effluent conductivity, which provides an operational indicator of dissolved ionic content and breakthrough behavior during demineralisation.^2^ Conductivity analysis provides an operational indication of ion leakage and overall demineralization performance. Final rinse conductivity was monitored until stabilization to <80 μS cm^–1^ before performance testing. Although direct measurements of pore structure, specific surface area, ion-exchange capacity, and intraparticle diffusion were not performed, the mechanistic interpretation presented herein is based on complementary physicochemical evidence from SEM, FTIR, particle size distribution, conductivity breakthrough, and ICP–MS analyses. Consequently, the proposed mechanism should be interpreted as an indirect physicochemical interpretation rather than a direct mechanistic confirmation.

Figure 6 presents the conductivity–time profiles for both UTR and PTR systems. For the UTR resin, a rapid decrease in effluent conductivity was observed within the first 15 min, indicating initial ion exchange, with hardness ions removed from the influent and captured by available active sites. However, after this initial phase, effluent conductivity gradually increased, returning to influent levels at approximately 49 min and then remaining stable. This trend indicates exhaustion of accessible exchange sites and the establishment of ion-exchange equilibrium, resulting in breakthrough behavior.^3^ The earlier exhaustion observed for UTR may be associated with differences in regeneration state and resin condition that influence ion transport and site accessibility. Such behavior is consistent with previous reports in which exhausted resins undergo morphological changes, including surface irregularity and partial pore blockage due to organic fouling, ultimately reducing treatment efficiency and operational lifespan.^15^ These limitations may hinder efficient utilization of the resin matrix during demineralization. In contrast, differences in surface deposits, morphology, and particle size observed following brine pretreatment may influence exchange-site accessibility and ion transport within the resin. These physicochemical differences may be associated with the longer observed demineralization period in the individual PTR column run.

**Figure 6:**
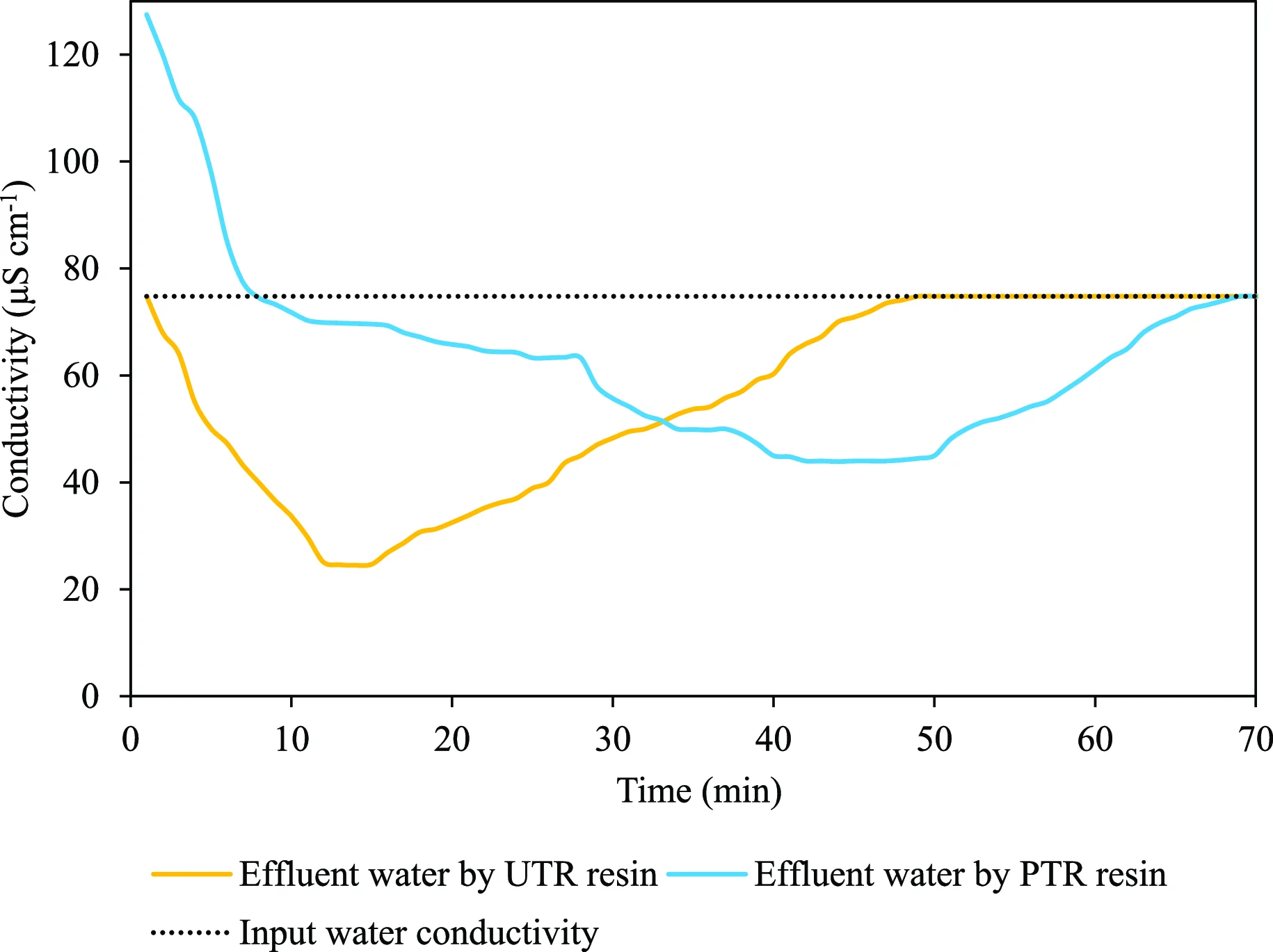
Conductivity (μS cm^–1^) as a function of time (min) during demineralization using UTR and PTR resins. Curves represent individual column runs (*n* = 1).

In contrast, the PTR resin exhibited an initial spike in conductivity during the first 8 min, exceeding the influent value. This early leakage phase may be associated with the release of loosely bound ions and residual salts remaining within the resin matrix following brine pretreatment, potentially involving ion diffusion from the hydrated resin matrix.^2^ Changes in resin hydration following regeneration may influence the internal ion mobility and contribute to the temporary increase in conductivity during the initial stage. Although this may result in a short-term increase in conductivity, the behavior may reflect transient ion release from the resin following brine pretreatment. However, the underlying transport mechanism has not been directly quantified. Additionally, organic fouling may contribute to premature ion release, particularly under dynamic flow conditions.^33^ From an operational perspective, this approximately 8 min initial leakage period represents a conditioning phase during which the effluent may not meet the required conductivity criteria. Where the applicable water-quality criterion is exceeded, this initial effluent would need to be diverted from the usable treated water stream until an acceptable conductivity is reached. Consequently, where the applicable conductivity criterion is exceeded, the initial leakage would reduce the net usable treated water volume of the PTR system. Additional rinsing or conditioning before column operation may help minimize residual ion release. However, this was not evaluated in the present study and requires further investigation.

The PTR resin maintained a lower conductivity for a longer period than the UTR resin, showing a longer low-conductivity period in the individual PTR run. However, a gradual increase in conductivity was observed beyond 57 min, which may reflect changes in column or ion-exchange behavior as exhaustion was approached. Despite this increase, the observed breakthrough time was longer for PTR than that for UTR in the individual column runs. The longer low-conductivity period observed for PTR was associated with a later resin exhaustion in this comparison.

To facilitate quantitative comparison of breakthrough behavior, the conductivity data were normalized using the influent conductivity (*C*_o_ = 74.8 μS cm^–1^), and the resulting breakthrough curves are presented as *C*/*C*_o_ versus time in Figure 7. Normalization reduces the influence of absolute conductivity values and facilitates comparison of breakthrough progression and exhaustion behavior. The breakthrough parameters derived from Figure 7 are summarized in Table 1. The breakthrough parameters presented in Table 1 were derived from the normalized breakthrough curves shown in Figure 7 and provide a quantitative comparison of resin utilization and exhaustion behavior between UTR and PTR systems.

**Figure 7:**
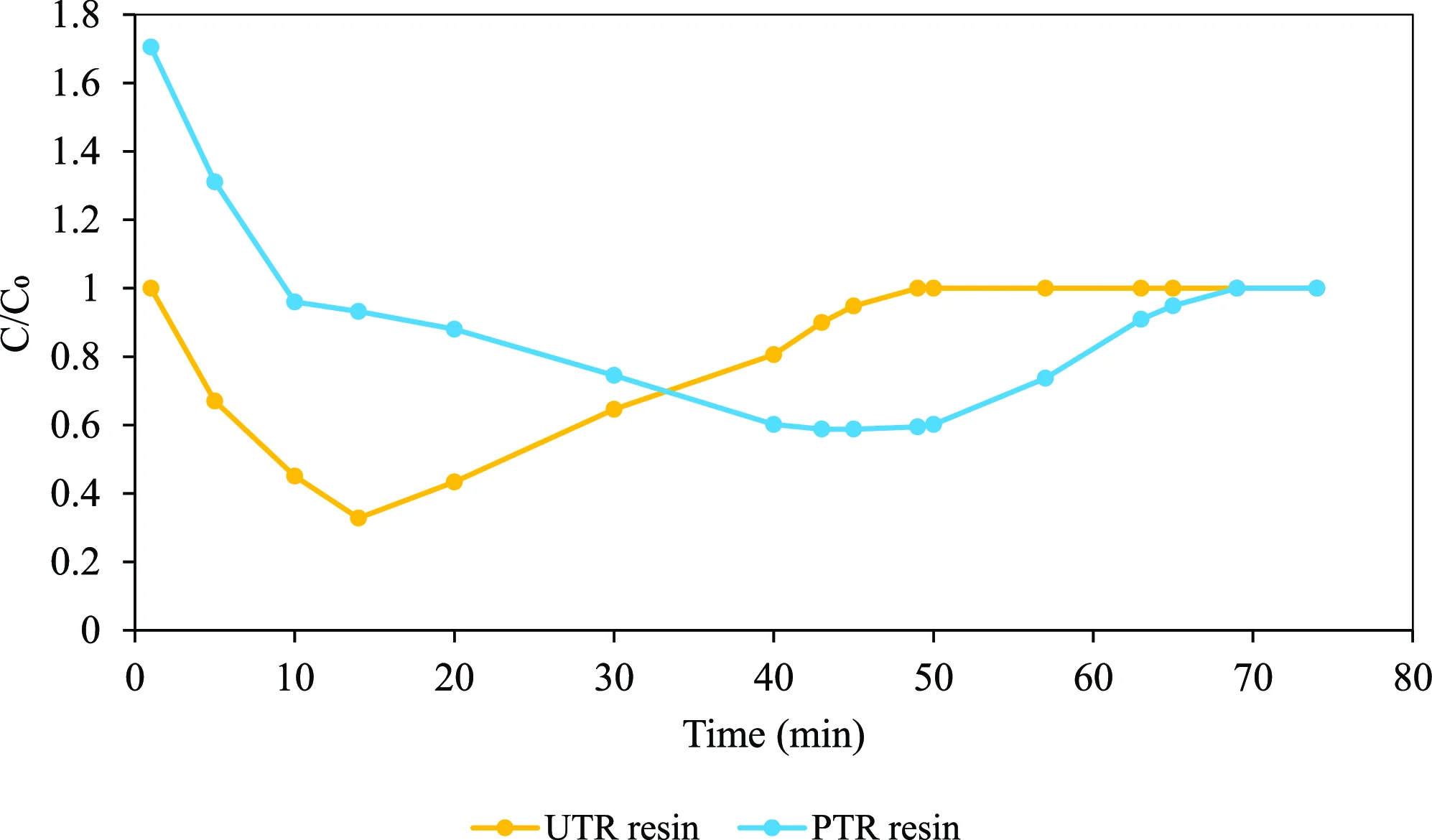
Normalized breakthrough curves (*C*/*C*_o_) during demineralization using UTR and PTR resins. Curves represent individual column runs (*n* = 1).

**Table 1:** Breakthrough Parameters for UTR and PTR Resins during Demineralization

parameter	UTR	PTR	observed increase (%)
exhaustion time (*C*/*C*_o_ = 1.0), min	49	69	40.8
time at *C*/*C*_o_ = 0.90, min	43	62.8	46
time at *C*/*C*_o_ = 0.95, min	45.1	65	44.1

Figure 7 presents the normalized breakthrough curves (*C*/*C*_o_) for UTR and PTR during the demineralization process. The individual breakthrough profiles showed a longer observed demineralization period and later exhaustion for PTR than UTR. For UTR, the *C*/*C*_o_ value decreased rapidly from 1.00 to a minimum of 0.328 within 14 min, indicating initial removal of dissolved ions from the influent. Thereafter, the *C*/*C*_o_ value increased progressively and reached 0.90, 0.95, and 1.00 at approximately 43, 45.1, and 49 min, respectively, indicating exhaustion of readily accessible exchange sites and reduced demineralization efficiency. In contrast, PTR showed a distinct breakthrough profile in Figure 7. Following the initial conditioning stage, the *C*/*C*_o_ value gradually decreased to a minimum of 0.588 at approximately 43–45 min and then increased toward exhaustion. Compared with UTR, PTR maintained lower *C*/*C*_o_ values for a longer period in the individual column run, corresponding to a later observed breakthrough. The *C*/*C*_o_ values of 0.90, 0.95, and 1.00 were reached at approximately 62.8, 65.0, and 69.0 min, respectively. In the individual column runs, the observed exhaustion time was 40.8% longer for PTR than UTR, while the observed times to reach *C*/*C*_o_ values of 0.90 and 0.95 were 46.0% and 44.1% longer, respectively. These individual column runs showed a longer observed resin utilization period and later exhaustion for PTR than UTR. The PTR individual column run exhibited a longer observed demineralization period and later exhaustion than the UTR run. The longer observed breakthrough times for PTR occurred alongside the physicochemical differences identified by SEM, FTIR, and particle-size analyses. However, exchange-site accessibility was not directly measured.

A substantial proportion of Ca^2+^ and Mg^2+^ was displaced during brine pretreatment before the subsequent acid–base regeneration step, while the individual PTR column run showed a longer observed resin utilization period than UTR. However, because the same acid and base regeneration conditions were used in the present study, a reduction in total acid or base consumption could not be directly demonstrated. Further optimization of regenerant dosage is required to determine whether the observed ion displacement during pretreatment can translate into lower overall chemical consumption. Although PTR resin exhibited a temporary increase in conductivity during the initial stage, potentially associated with residual salt release, its demineralization period in the individual PTR run was longer than that of UTR, indicating that brine pretreatment warrants further evaluation as a regeneration pretreatment strategy. Future studies incorporating porosimetry, ion-exchange capacity measurements, diffusion analysis, and multicycle regeneration experiments are needed to further evaluate the proposed mechanism.

### 3.7 ICP–MS Evaluation of Ion Removal and the Contribution of Brine Pretreatment

Figure 8 illustrates the removal performance of Mg, Ca, and Fe from residual ions in the brine and regeneration solutions for both UTR and PTR resins. Total Mg^2+^ removal was 25,941 ppb for PTR, substantially higher than the 8049 ppb observed for UTR. Notably, most Mg^2+^ was displaced during brine pretreatment in PTR, with 17,212 ppb detected in the spent brine and 8729 ppb in the HCl regenerant. In UTR, Mg^2+^ displacement relied exclusively on the chemical regeneration step. Approximately 66.4% of Mg^2+^ displaced from PTR was detected in the spent brine, indicating that a substantial proportion of Mg^2+^ displacement occurred before the subsequent acid regeneration step.

**Figure 8:**
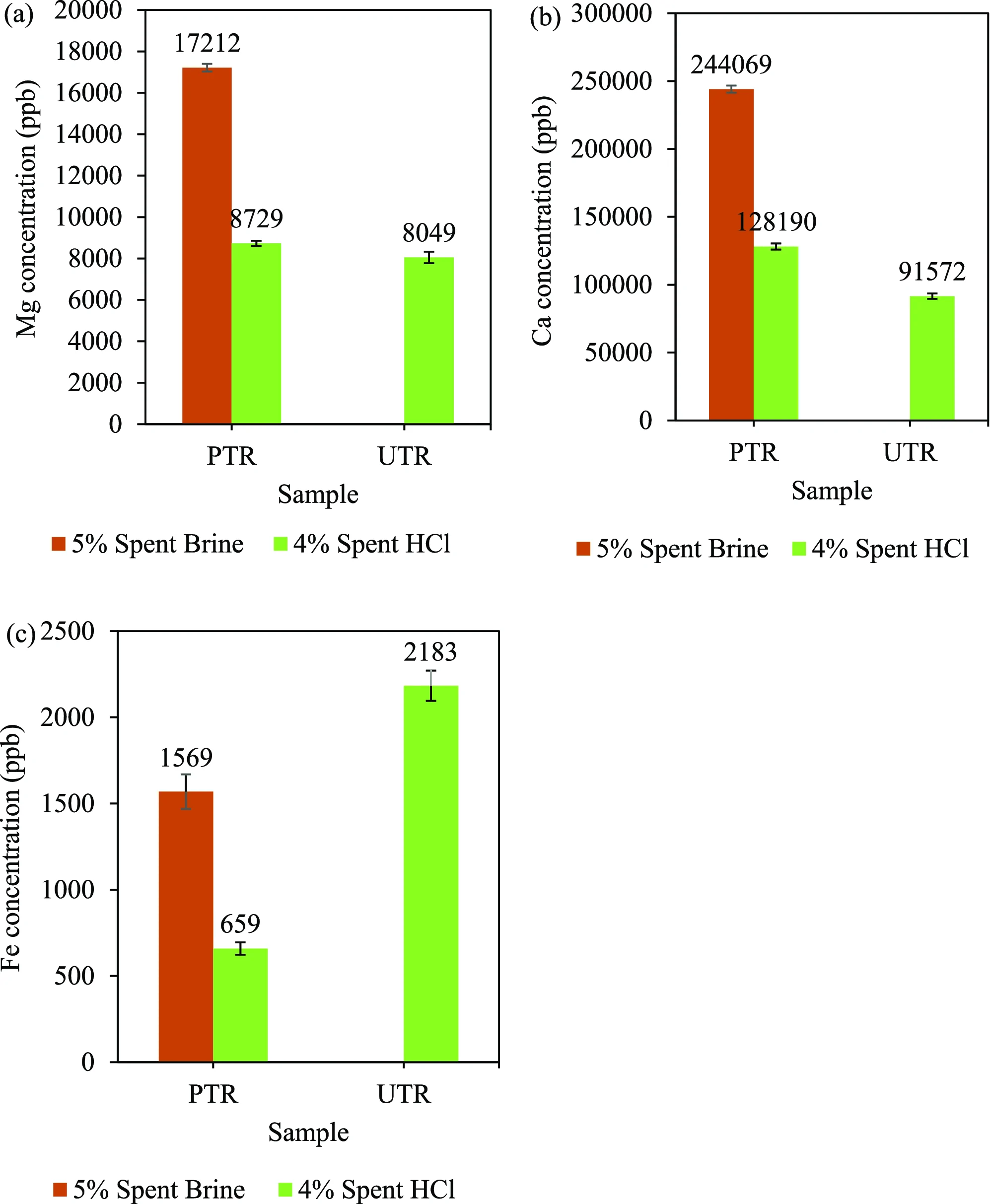
Residual (a) Mg, (b) Ca, and (c) Fe concentrations in the brine and regenerant solutions of the UTR and PTR resins. Results represent the mean ± standard deviation (*n* = 3).

A larger difference between PTR and UTR was observed for Ca removal. PTR resin achieved a total Ca^2+^ removal of 372,260 ppb, including 244,070 ppb from spent brine and 128,190 ppb from HCl regenerant, while UTR resin removed only 91,572 ppb via HCl regeneration. This represents approximately 306% higher total Ca^2+^ removal for PTR than UTR and is consistent with previous reports on Ca^2+^ removal using multistep regeneration strategies.^21,26,34^ Approximately 65.6% of the Ca^2+^ displaced from PTR was detected during brine pretreatment, indicating that substantial Ca^2+^ displacement occurred before acid treatment. The substantial displacement of Ca^2+^ and Mg^2+^ during the pretreatment stage indicates that a considerable proportion of these ions was displaced before subsequent chemical regeneration. Whether this permits lower strong-regenerant requirements remains to be evaluated through further process optimization. Staged and segmented regeneration strategies have previously been investigated as approaches to improve regenerant utilization and reduce chemical consumption while maintaining regeneration performance.^4,13^ In the present study, 5% NaCl achieved substantial ion displacement at a lower brine concentration than the 8–12% NaCl conditions reported for high-concentration regeneration strategies. This finding suggests that optimizing pretreatment conditions may provide a balance between ion displacement and the use of lower-concentration brine.

Fe removal showed minimal differences between PTR and UTR, with PTR achieving 2228 ppb and UTR 2 183 ppb (Figure 8c). This may be related to the low Fe concentration and differences in its interaction with the resin compared to the divalent ions examined. The difference in Fe removal between PTR and UTR was approximately 2% under the conditions evaluated.

Overall, most Ca^2+^ and Mg^2+^ displacement from PTR resin occurred during brine pretreatment, indicating that a substantial proportion of these ions was displaced before subsequent chemical regeneration. However, the present study did not directly evaluate reductions in the acid or base dosage. Therefore, any reduction in overall chemical consumption remains to be established through further process optimization. The FTIR and SEM observations indicated changes in surface-associated species and morphology following pretreatment, which were consistent with the observed differences in regeneration performance. The observed physicochemical changes may be related to exchange site accessibility and were accompanied by higher Ca^2+^ and Mg^2+^ removal under the conditions evaluated in this study. This is particularly important because strong-base IERs can retain more than 90% of their functional capacity over repeated regeneration cycles when fouling is controlled, while organic fouling remains the primary cause of performance deterioration.^35^

The observed differences following brine pretreatment may involve several complementary processes. The high Na^+^ concentration may promote the displacement of retained Ca^2+^ and Mg^2+^ through mass-action effects before subsequent acid–base treatment. This is supported by the ICP–MS results, which show that 65.6% of Ca^2+^ and 66.4% of Mg^2+^ were displaced during brine pretreatment. In addition, NaCl exposure may influence osmotic swelling of the resin matrix, which could affect polymer chain mobility and ion transport. SEM observations also suggest differences in surface-associated deposits, which may influence access to exchange sites. Similar recent studies have emphasized that resin regeneration performance depends strongly on ion accessibility, diffusion pathways, and the controlled use of regenerants. However, because pore structure, specific surface area, ion-exchange capacity, and intraparticle diffusion coefficients were not measured directly, these mechanisms should be interpreted as indirect physicochemical evidence rather than as direct mechanistic confirmation.

Table 2 summarizes established and emerging ion-exchange regeneration strategies, highlighting their operational characteristics, performance trade-offs, and implications for regeneration efficiency, as reviewed by Liu et al.^4^ Conventional acid–base regeneration remains effective and widely used. However, it is associated with high chemical consumption and the generation of corrosive waste streams.^6,20^ NaCl brine pretreatment has been investigated as an alternative regeneration approach, but most studies rely on high salt concentrations or single-parameter optimization, with limited consideration of the relationships among process conditions, resin structure, and regeneration performance.^4^ Consequently, the mechanisms governing ion transport and accessibility remain insufficiently understood. Alternative regenerants and chemical-free approaches have been explored,^36–39^ but their broader application is constrained by cost, operational complexity, and scalability. Similarly, while brine management strategies can reduce waste, they often introduce performance trade-offs over repeated cycles.^4,40^ In contrast, this study applies previously optimized NaCl brine pretreatment conditions (5% NaCl, 1:1 resin-to-brine ratio, 55 °C) as a controlled pretreatment before regeneration. In the present experiments, substantial Ca^2+^ and Mg^2+^ displacement occurred during pretreatment, while physicochemical observations were potentially related to exchange-site accessibility, and the individual PTR column run showed a longer resin-utilization period. The results provide indirect physicochemical evidence linking brine pretreatment with the observed changes in resin properties and regeneration performance.

**Table 2:** Comparison of Literature-Reported Ion-Exchange Regeneration Strategies and Their Implications for Regeneration Efficiency and Brine Management^a^

regeneration strategy	typical conditions	key features	limitations	key insight	references
conventional acid–base regeneration	HCl (∼4%) and NaOH (∼4%)	high efficiency; widely used	high chemical use; corrosive waste	chemically intensive baseline	^6,20^
high-concentration NaCl regeneration	8–12% NaCl brine	simple; cost-effective	high-salinity waste; disposal issues	major source of brine burden	^4^
segmented or optimized regeneration	multistep NaCl (e.g., staged low/high concentration)	better utilization; reduced brine	process control required; case-specific	highlights the need for optimization	^41,42^
alternative regenerants	KCl, NaHCO_3_, AlCl_3_	lower environmental impact; resource recovery potential	higher cost; lower efficiency	sustainability–performance trade-off	^38,39^
chemical-free regeneration approaches	biological, electrochemical, thermal methods	no chemical input	complex; limited scalability	promising but not yet practical	^36,37^
brine management strategies	reuse cycles, membrane treatment, crystallization	reduces salt use and waste	performance decline; extra treatment	key sustainability bottleneck	^4,40^
optimized NaCl brine pretreatment integrated with regeneration (this study)	5% NaCl, 1:1 resin-to-brine ratio, 55 °C, followed by regeneration	substantial Ca^2+^ and Mg^2+^ displacement during pretreatment; physicochemical changes potentially related to exchange-site accessibility; longer observed resin utilization in the individual PTR column run	requires controlled operating conditions; slight initial ion leakage may occur	optimized conditions associated with physicochemical changes and regeneration performance in the present study	^17^ and this study

a The conditions presented for conventional acid–base regeneration represent typical conditions reported in the cited literature and differ from the experimental conditions used in the present study (1% NaOH and 4% HCl).

A comparison of chemical and water requirements for UTR and PTR regeneration is presented in Table 3. Both resins were subjected to the same subsequent acid–base regeneration conditions, using 36 mL of 4% (w/v) HCl and 16 mL of 1% (w/v) NaOH. Both UTR and PTR also required 700 mL of DI water for backwashing before resin separation and chemical regeneration. PTR additionally required 30 mL of 5% (w/v) NaCl, 5.5 L of DI water for pretreatment rinsing and soaking, and 0.5 L of tap water for final conditioning, generating approximately 30 mL of spent brine. Thus, although substantial Ca^2+^ and Mg^2+^ displacement occurred during brine pretreatment before subsequent acid–base regeneration, the present study did not demonstrate a reduction in total chemical or water consumption. Further optimization of regenerant dosage and rinsing requirements is necessary to determine whether this approach can provide an overall reduction in chemical and water use.

**Table 3:** Comparison of Chemical Use, Water Requirements, and Spent-Brine Generation for UTR and PTR Regeneration in the Present Study

parameter	UTR	PTR
NaCl pretreatment	none	30 mL of 5% (w/v) NaCl
NaOH regeneration	16 mL, 1% (w/v)	16 mL, 1% (w/v)
HCl regeneration	36 mL, 4% (w/v)	36 mL, 4% (w/v)
backwash water	700 mL of DI water	700 mL of DI water
pretreatment rinse/conditioning water	none	5.5 L of DI water +0.5 L of tap water
spent brine generated	none	∼30 mL

The present study evaluated only one regeneration cycle. Therefore, the effectiveness of brine pretreatment over repeated regeneration cycles remains to be established. Future multicycle studies should evaluate regeneration efficiency, resin stability, chemical consumption, rinsewater requirements, and waste-brine generation to determine the long-term operational and environmental benefits of the proposed approach.

## 4 Conclusions

This study examined the association between controlled brine pretreatment, resin physicochemical characteristics, and subsequent regeneration performance. Under the previously optimized conditions of 5% NaCl, a 1:1 resin-to-brine ratio, and 55 °C, pretreated resins achieved approximately 306% higher Ca^2+^ removal and 222% higher Mg^2+^ removal than untreated regenerated resins. In individual column runs, PTR exhibited a longer observed demineralization period, with exhaustion occurring at 69 min compared with 49 min for UTR. SEM showed no obvious surface-associated deposits on the pretreated resin, while FTIR analysis indicated minor spectral changes with characteristic resin functional groups largely retained. Particle-size analysis showed a shift toward smaller particle sizes after brine pretreatment. ICP–MS analysis further showed that approximately two-thirds of the Ca^2+^ and Mg^2+^ removed from PTR were displaced during the pretreatment stage before subsequent acid–base regeneration. Collectively, these findings provide indirect physicochemical evidence that the observed changes in resin characteristics may influence ion transport and the accessibility of exchange sites. However, direct measurements of pore structure, surface area, ion-exchange capacity, and ion diffusion were not performed, and further studies incorporating such measurements are warranted to verify the proposed mechanism. Therefore, controlled brine pretreatment shows potential as a regeneration pretreatment strategy.

## Data Availability

The data supporting the findings of this study are not publicly available due to their relevance to ongoing and unpublished work but are available from the corresponding author upon reasonable request.
